# Supplementary material for: Macrophages self-generate and refine chemotactic gradients during migration towards complement C5a
Source: PLoS Biol. 2026 Apr 2;24(4):e3003728. doi: 10.1371/journal.pbio.3003728 (PMC13061319; doi:10.1371/journal.pbio.3003728)
Supplement: S1 Fig — The red dashed box highlights the leading cell wave. Solid black and yellow lines indicate the distance covered by cells on the bridge at low and high concentrations, respectively. (PDF) [file pbio.3003728.s001.pdf]

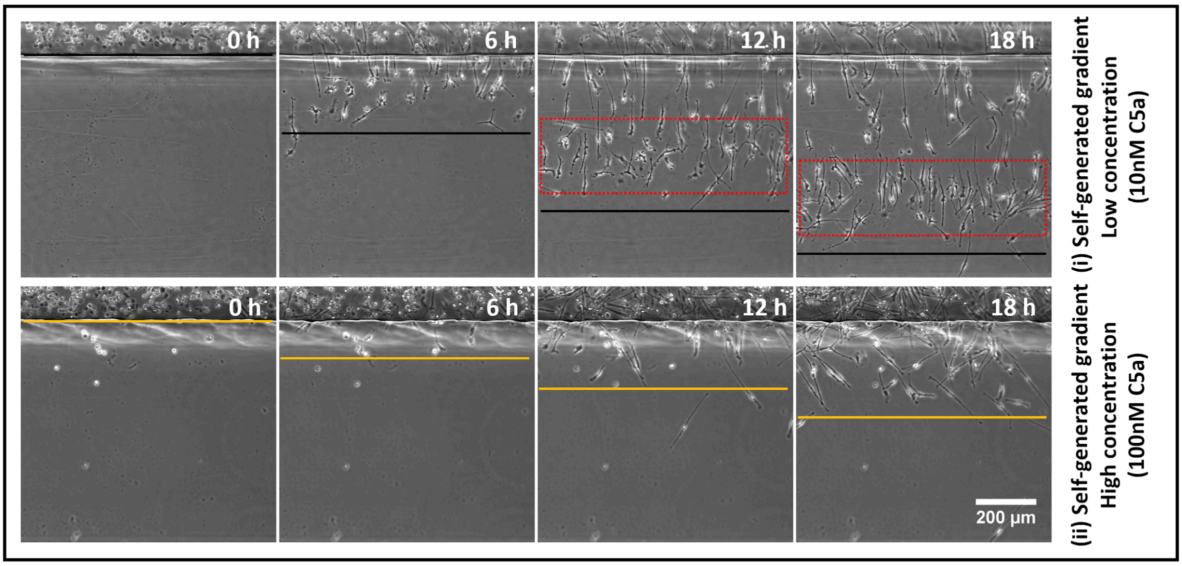

Figure S1 - Self-generated gradient responses at (i) 10 nM C5a and (ii) 100 nM C5a. Red dashed box highlights the leading cell wave. Solid black and yellow lines indicate the distance covered by cells on the bridge at low and high concentrations, respectively.
