# Supplementary material for: Macrophages self-generate and refine chemotactic gradients during migration towards complement C5a
Source: PLoS Biol. 2026 Apr 2;24(4):e3003728. doi: 10.1371/journal.pbio.3003728 (PMC13061319; doi:10.1371/journal.pbio.3003728)
Supplement: S2 Fig — The migration response of BMDM cells seeded at (A) High cell density (6 h), (B) Medium cell density (12 h), and (C) Low cell density (24 h) for different uniform C5a dose. (PDF) [file pbio.3003728.s002.pdf]

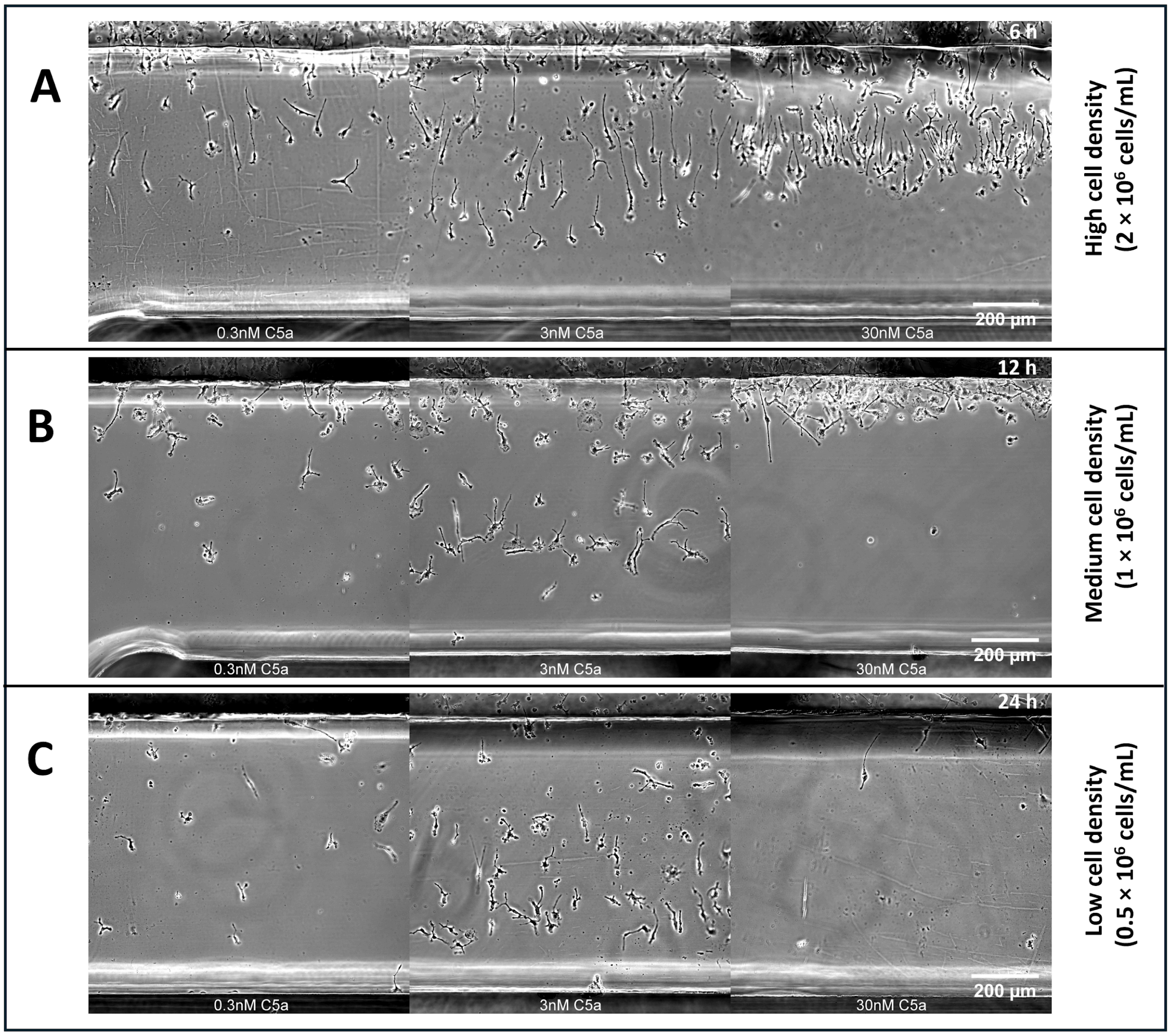

Figure S2 - Effect of initial cell density on C5a dose response. The migration response of BMDM cells seeded at (A) High cell density (6 h), (B) Medium cell density (12 h), and (C) Low cell density (24 h) for different uniform C5a dose.
