# Supplementary material for: Macrophages self-generate and refine chemotactic gradients during migration towards complement C5a
Source: PLoS Biol. 2026 Apr 2;24(4):e3003728. doi: 10.1371/journal.pbio.3003728 (PMC13061319; doi:10.1371/journal.pbio.3003728)
Supplement: S3 Fig — C5a-des-R was seen under all conditions tested. Mouse BMDMs under similar conditions showed no trimming of mC5a. (PDF) [file pbio.3003728.s003.pdf]

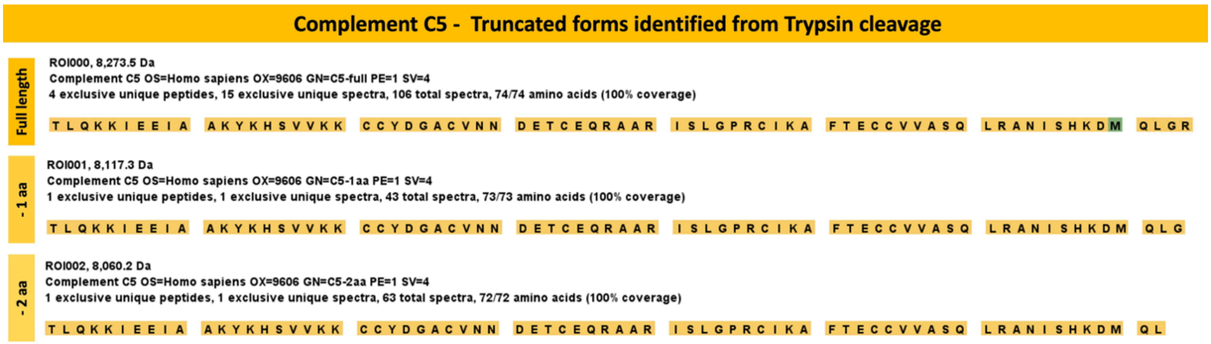

Figure S3 - Mass spectrometry report for C5a trimming by cultured human THP-1 macrophages incubated with hC5a. C5a-des-R was seen under all conditions tested. Mouse BMDMs under similar conditions showed no trimming of mC5a.
