# Supplementary material for: Macrophages self-generate and refine chemotactic gradients during migration towards complement C5a
Source: PLoS Biol. 2026 Apr 2;24(4):e3003728. doi: 10.1371/journal.pbio.3003728 (PMC13061319; doi:10.1371/journal.pbio.3003728)
Supplement: S1 Table — (DOCX) [file pbio.3003728.s004.docx]

Submitted Manuscript: Confidential Template updated February 2021

| Parameter | Value |
| --- | --- |
| Diffusion of C5a | 100 μm2/second |
| Cell diameter | 30 μm |
| Cell speed | 0.8 μm/minute |
| C5a receptor Kd | 2 nM |
| Maximum C5a endocytosis per cell | 3.69x10-11 nmol/minute |

S1 Table. Parameters of computational model.
