# Supplementary material for: Macrophages self-generate and refine chemotactic gradients during migration towards complement C5a
Source: PLoS Biol. 2026 Apr 2;24(4):e3003728. doi: 10.1371/journal.pbio.3003728 (PMC13061319; doi:10.1371/journal.pbio.3003728)
Supplement: Attachment — Submitted filename: Response to reviewers.docx [file pbio.3003728.s015.docx]

Dear Editors,

We were very grateful for these referee reports – the work they asked for has consistently strengthened the paper. Please thank them direct from us.

A detailed response is given below, but in general we have done everything they asked.

Rev. 1:
The authors of this enlightening study have demonstrated that macrophages can perform self-generated gradient chemotaxis towards C5a, turning a flat concentration of C5a into a gradient, and then migrating up that gradient. They also showed that normal chemotaxis requires the same self-generated gradient. They confirmed that there is an optimal concentration of C5a. Experiments and analysis indicate that BMDMs use clathrin-mediated endocytosis for the C5a uptake.

Thanks for this assessment.

The paper is clearly written. Here is what can improve it:

1) the model was used for the initial result in fig 1, and was not applied to anything else in the paper. It could be useful, for example, to simulate the results of fig 3.

We have now included a second panel in Fig. 1, and a movie is included as movie 6. The model works nicely here too.

2) The 'wave of cell migration' is just a visual impression for now. it would be better to quantify this phenomenon and report quantitative measures.

This is a very good point. We initially included it as supplementary, but on reflection it strengthens the argument so we added it to Fig. 1 and moved other data instead.

3) It would be interesting to try to change the initial cell densities and measure responses, as well as simulating such experiments with the model.

This has now been added as supplementary fig 2. It makes surprisingly little difference, because the wave is self-sizing; the only substantive change is the time taken for the wave to start. It is a point we have studied in depth in past work, see Tweedy et al 2016.

4) Quantifying a response to not only the flat concentration of C5a but also to an initial gradient of C5a would be informative.

Quantification of an imposed gradient of C5a, observed in experiment Fig 1B(i) ,is shown in Supplement Figure S1B and its simulation shown in Fig 1 C and Movie 2.


Rev. 2:
Phagocytes are guided to sites of inflammation by complement C3a and C5a, as well as other chemoattractants. It has been known for a long time that membrane-bound enzymes can degrade extracellular chemoattractants, such as cAMP (PDE expressed in Dictyostelium) (e.g. Sucgang et al., Dev. Biol. 1997) and complement C5a (carboxypeptidase M in immune cells) (e.g. Rehli et al. 1995; Krause et al., Immunol. Rev. 1998), and thereby reshape the chemoattractant gradient. However, both enzymatic degradation and receptor-mediated internalization of chemoattractants have emerged as functionally important mechanisms of self-generated gradients (e.g. Boldajipour et al., Cell 2007; Dona et al., Nature 2013; Tweedy et al., PloS Biol. 2016; Tweedy & Insall, Front. Cell Dev. Biol. 2020), allowing chemotaxis in the absence of an imposed gradient (i.e. in a uniform concentration) or enhancing directional migration in the presence of a defined concentration gradient.

In this study, Kiran et al. nicely demonstrated that both mouse bone marrow-derived macrophages (BMDMs) and a human myeloid cell line (THP-1 cells) can self-generate a chemoattractant gradient, albeit using differents mechanisms. Using a fluorescent ligand, the authors could show that mouse BMDMs self-generate a gradient through complement C5a receptor 1 (C5aR1)-mediated complement C5a internalization. In contrast, THP-1 cells, but not BMDMs, degraded complement C5a through the activity of membrane-bound carboxypeptidase M (confirmed by mass spectrometry). Moreover, maximal cell density at the wave front of self-generated chemotaxis was observed at higher starting complement C5a concentrations, which could be explained by the higher rate of chemoattractant depletion by enzymatic activity compared to receptor-mediated internalization and receptor recycling. That is, mouse BMDMs, which lack the enzyme, are limited by receptor availability. The authors additionally showed,
using BMDMs, that pretreatment with a Toll-like receptor 4 ligand, lipopolysacchardies (LPS), augmented chemotaxis.

The experiments are well designed and the data nicely support the conclusions. This study advances the concept of self-generated gradients as a key element in chemotactic signaling and offers a framework to both interpret and predict the behavior of motile immune cells.

Thanks for the positive assessment. We have included the following citations:

Rehli et al. 1995; Krause et al., Immunol. Rev. 1998; Boldajipour et al., Cell 2007; Dona et al., Nature 2013

Comments
1. The authors should discuss why THP-1 cells (human monocytic leukemia line) were used instead of, for example, human peripheral blood monocyte-derived macrophages.

We have added a note in the text. We would like to use primary human cells, but lack the capacity at present. This point has goaded us to find competent collaborators for a future attempt.

2. Please discuss the differences in actin cytoskeletal dynamics in mouse BMDMs versus THP-1 cells. The former appear to have low rear end RhoA/actomyosin activity, whereas the latter exhibit a rounded morphology.

An extremely interesting point, but hard to fit into the text – we were only really interested in the dose response, which is independent of the mechanism of migration.

3. The authors should briefly discuss the potential roles of relay signals, such as auto- and paracrine Leukotriene B4 (LTB4) and ATP signaling in self-generated gradients and chemotaxis.

This is an _extremely_ interesting point. We (of course) looked. Mass spectrometry showed no LTB4 at all (but like all negative results, hard to control). We included apyrase to remove ATP, but it made cells chemotax much faster, in a slightly nonreproducible way (sometimes a bit faster, sometimes a lot). We are not averse to including some of this data if the referee really wants, but it’s confusing and doesn’t help interpret the results we see.

We included a line in the text about secondary attractants.

Minor comments
1. New Figure 1: It would be helpful to include a new figure (Figure 1), which shows the Insall chamber in a schematic form. This will help explain the lateral gray-shaded structures in some of the figures and supplementary movies, e.g. Fig. 1C, and save the "busy reader" from looking up the original description of the chamber.

Good idea. Thanks. Done.

2. Abstract: The word "enzymatic" is perhaps superfluous in the text "through carboxypeptidase-mediated enzymatic degradation"?

Correct. Thanks. Removed.

3. Figure 1C: It would help the reader if "Self-generated gradient" was included in the label at the end of end panel. Otherwise, the "busy reader" needs to consult the legend.

Good idea. Thanks. Done.

We have made the other minor corrections suggested below point 3, also with thanks.
