# Supplementary material for: Macrophages self-generate and refine chemotactic gradients during migration towards complement C5a
Source: PLoS Biol. 2026 Apr 2;24(4):e3003728. doi: 10.1371/journal.pbio.3003728 (PMC13061319; doi:10.1371/journal.pbio.3003728)
Supplement: Attachment — Submitted filename: Final changes.docx [file pbio.3003728.s016.docx]

Changes

Title changed to “Macrophages Self-Generate And Refine Chemotactic Gradients During Migration Towards Complement C5a”. The suggestion about refinement is good, and this title is much tighter. It’s the gradients more than the chemotaxis per se that are enhanced. And very, very few paper titles refer to “complement component 5a” – ChatGPT says 3% - so it seems rather arch. I hope this meets the spirit of the request.

The original data were provided and referenced but not directly in the figure legend. We have now referred directly to them in each figure legend. I hope this is what you were after. If not, please say exactly what is wrong and we will address it. We have also permanently archived the code as requested.

The financial statement has been enlarged (I’m not 100% certain what was missing, maybe just format quality, but if something else is please say exactly what).
